# Integrated information as a common signature of dynamical and information-processing complexity

**DOI:** 10.1063/5.0063384

**Published:** 2022-01-01

**Authors:** Pedro A. M. Mediano, Fernando E. Rosas, Juan Carlos Farah, Murray Shanahan, Daniel Bor, Adam B. Barrett

**Affiliations:** 1 Department of Psychology, University of Cambridge, Cambridge CB2 3EB, United Kingdom; 2 Centre for Psychedelic Research, Department of Brain Science, Imperial College London, London SW7 2DD, United Kingdom; 3 Data Science Institute, Imperial College London, London SW7 2AZ, United Kingdom; 4 Centre for Complexity Science, Imperial College London, London SW7 2AZ, United Kingdom; 5 School of Engineering, École Polytechnique Fédérale de Lausanne, CH-1015 Lausanne, Switzerland; 6 Department of Computing, Imperial College London, London SW7 2RH, United Kingdom; 7 Sackler Center for Consciousness Science, Department of Informatics, University of Sussex, Brighton BN1 9RH, United Kingdom; 8 The Data Intensive Science Centre, Department of Physics and Astronomy, University of Sussex, Brighton BN1 9QH, United Kingdom

## Abstract

The apparent dichotomy between information-processing and dynamical approaches to complexity science forces researchers to choose between two diverging sets of tools and explanations, creating conflict and often hindering scientific progress. Nonetheless, given the shared theoretical goals between both approaches, it is reasonable to conjecture the existence of underlying common signatures that capture interesting behavior in both dynamical and information-processing systems. Here, we argue that a pragmatic use of integrated information theory (IIT), originally conceived in theoretical neuroscience, can provide a potential unifying framework to study complexity in general multivariate systems. By leveraging metrics put forward by the integrated information decomposition framework, our results reveal that integrated information can effectively capture surprisingly heterogeneous signatures of complexity—including metastability and criticality in networks of coupled oscillators as well as distributed computation and emergent stable particles in cellular automata—without relying on idiosyncratic, *ad hoc* criteria. These results show how an agnostic use of IIT can provide important steps toward bridging the gap between informational and dynamical approaches to complex systems.

## I Introduction

Most theories about complexity are rooted in either information theory or dynamical systems perspectives—two disciplines with very different aims and toolkits. The former, built after the work of Turing and Shannon, focuses mainly on discrete systems and considers complexity in terms of information processing, universal computation, distributed computation, and coherent emergent structures.^1^ The latter, following the tradition started by Poincaré and Birkhoff, focuses on continuous systems and studies their behavior using attractors, phase transitions, chaos, and metastability.^2^

This methodological divide has contraposed how various communities of researchers think about complex systems, even to the extent of triggering some longstanding disagreements. This is particularly evident in the field of cognitive neuroscience, where proponents of computational approaches claim that the brain works similarly to a Turing machine,^3–6^ while opponents believe that cognitive processes are essentially continuous and rate-dependent.^7–9^ A related debate has taken place in the artificial intelligence community between symbolic and connectionist paradigms for the design of intelligent systems.^10^ Modern stances on these problems have promoted hybrid approaches,^11^ bypassing ontological arguments toward epistemological perspectives where both information and dynamics represent equally valid methods for enquiry.^12^

Interestingly, bridging the gap between the informationprocessing and dynamical systems literature has proven scientifically fruitful. Examples of this are Wolfram’s categorization of cellular automata in terms of their attractors, which provided insights into the possible types of distributed computation enabled by these systems according to dynamicalproperties of their trajectories,^13^ and Langton’s intuition that computation takes place in a phase transition “at the edge of chaos.”^14^ This rich point of contact, in turn, suggests that what informational and dynamical approaches deem as interesting might have a common source, beyond the apparent dissimilarities introduced by heterogeneous tools and disciplinary boundaries.

In this article, we propose *Integrated Information Theory* (IIT),^15–17^ together with its recent extension *Integrated Information Decomposition* (ΦID),^18^ as a conceptual framework that can help bridge the gap between information-processing and dynamical systems approaches. At its inception, IIT was a theoretical effort that attempts to explain the origins and fundamental nature of consciousness.^19,20^ The boldness of IIT’s claims has not gone unnoticed, and they have caused a heated debate in the neuroscience community.^21–23^ Unfortunately, its audacious claims about consciousness have kept many scientists away from IIT, thereby preventing some of its valuable theoretical insights from reaching other areas of knowledge.

We advocate for the adoption of a *pragmatic* IIT, which can be used to analyze and understand complex systems without the philosophical burden of its origins in consciousness science. Consequently, the goal of this paper is to dissociate IIT’s claims as a theory of consciousness from its formal contributions and put the latter to use in the context of complexity science. For this purpose, we demonstrate that integrated information—as calculated in ΦID—peaks sharply for oscillatory dynamical systems that exhibit criticality and metastability and also characterizes cellular automata that display distributed computation via persistent emergent structures. These findings illustrate the remarkable flexibility of integrated information measures in discovering features of interest in a wide range of scenarios, without relying on domain-specific considerations. Overall, this work reveals how a grounded, demystified interpretation of IIT can allow us to identify features that are transversal across complex information-processing and dynamical systems.

The rest of this article is structured as follows. Section II presents the core formal ideas of IIT and ΦID in a simple manner and puts forward a sound and demystified way of interpreting its key quantities. Our main results are presented in Secs. III and IV: the former shows that integrated information can capture metastability and criticality in dynamical systems, and the latter that integrated information is a distinctive feature of distributed computation. Finally, Sec. V discusses the significance of these results and summarizes our main conclusions.

## II A Pragmatist’s IIT

IIT constitutes one of the first attempts to formalize what makes a system “more than the sum of its parts,” building on intuitive notions of synergy and emergence that have been at the core of complexity science since its origins.^24,25^ IIT proposes *integrated information* for that role, informally defining it as information that is contained in the interactions between the parts of a system and not within the parts themselves. The core element of IIT is Φ, a scalar measure that accounts for the amount of integrated information present in a given system.

While faithful to its original aims, throughout its life, IIT has undergone multiple revisions. Of all of them, we will focus on the theory as introduced by Balduzzi and Tononi in 2008.^16^ While more recent accounts of the theory exist,^17^ these place a much stronger emphasis on its goals as a theory of consciousness, at the expense of a departure from standard information-theoretic practice and more convoluted algorithms—which have hindered its reach and made the theory applicable only in small discrete systems.

### A The maths behind Φ

**Fig. 1:**
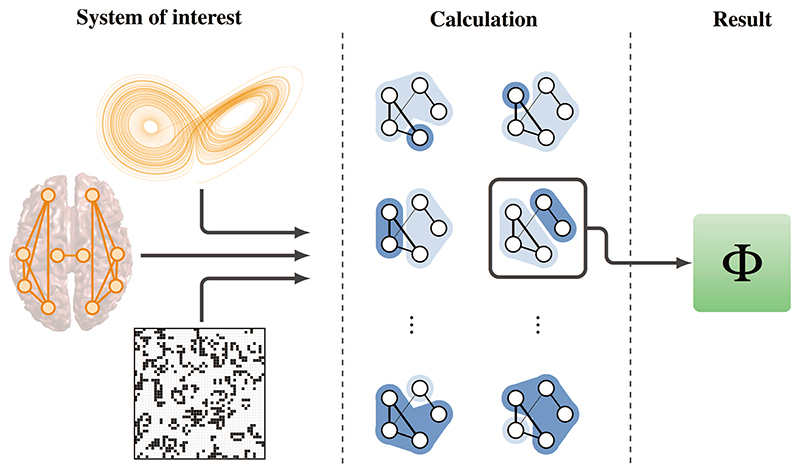
Graphical illustration of an integrated information analysis in complex systems. Integrated information theory (IIT) can be used to analyze a wide range of complex systems from dynamical systems to cellular automata and empirical data (left). Due to the generality of information-theoretic measures, it can be applied to either real- or discrete-valued data. After a suitable statistical model has been estimated, the system is partitioned following Eq. (2), and effective information *φ* is computed for each partition (middle). Finally, the partition with the “cruelest cut” (more formally, the minimum information partition) is selected, and the final value of integrated information Φ is computed (right).

This section provides a succinct description of the mathematical formulas behind IIT 2.0,^16^ following Barrett and Seth’s^26^ concept of *empirical* integrated information. The overall analysis procedure is represented schematically in Fig. 1.

The building block of integrated information is a measure of *effective information* (i.e., excess of predictive information) typically denoted as *φ*.^16^ Effective information quantifies how much better a system *X* is at predicting its own future after a time *τ* when it is considered a whole compared to when it is considered the sum of two subsystems *M*^1^ and *M*^2^ [so that *X* = (*M*^1^, *M*^2^)]. In other words, *φ* evaluates how much predictive information is generated by the system over and above the predictive information generated by the two subsystems alone. For a given bipartition $B=\{M^{1},M^{2}\},$ the effective information of the system *X* beyond B is calculated as

(1) $$\varphi [X;\tau ,B]=I(X_{t-\tau };X_{t})-\sum_{k=1}^{2}I(M_{t-\tau }^{k};M_{t}^{k}),$$

where *I* is Shannon’s mutual information. We refer to *τ* as the *integration timescale*.^27^

The core idea behind the computation of Φ is to (i) exhaustively search all possible partitions of the system, (ii) calculate *φ* for each of them, and (iii) select the partition with lowest *φ* (under some considerations, see below), termed the minimum information bipartition (MIB). Then, the integrated information of the system is defined as the effective information beyond its MIB. Concretely, the integrated information Φ associated with the system *X* over the integration scale *τ* is given by

(2a) $$\Phi [X;\;\tau ]=\varphi [X;\;\tau ,\;B^{\text{MIB}}],$$

(2b) $$B^{\text{MIB}}=\arg_{B}\min\frac{\varphi [X;\;\tau ,\;B]}{k(B)},$$

(2c) $$K(B)=\min\;\{H(M^{1}),\;H(M^{2})\},$$

where *K* is a normalization factor introduced to avoid biasing Φ to excessively unbalanced bipartitions. Defined this way, Φ can be understood as the minimum information loss incurred by considering the whole system as two separate subsystems.

An important drawback of Φ is that it can take negative values, which hinders its interpretation as a measure of system-wide integration.^28^ Recently, Mediano *et al*.^18^ showed that Φ quantifies not only information shared across and transferred between the parts, but also includes a negative component measuring *redun-dancy*—i.e., when the parts contain *the same* predictive information. Therefore, Φ measures a balance between information transfer and redundancy such that Φ < 0 when the system is redundancy-dominated.

A principled way to address this limitation is to refine Φ by disentangling the different information phenomena that drive it. This can be achieved via the ΦID framework,^18^ which provides a taxonomy of “modes” of information dynamics in multivariate dynamical systems. Using ΦID, one can define a revised version of *φ*, denoted as *φ*^R^,^18^ which removes the negative redundancy component in Φ by simply adding it back in. Work is ongoing on understanding different possible redundancy functions. Here, we compute *φ*^R^ via the minimum mutual information (MMI)^29^ redundancy function, which yields

(3) $$\varphi^{\text{R}}[X;\;\tau ,\;B]=\varphi [X;\;\tau ,\;B]\;+\;\underset{i,j}{\min}\;I(M_{t-\tau }^{i};M_{t}^{j}).$$

Using *φ*^R^, we can define Φ^R^ analogously through Eq. (2).

Note that this revised measure of integrated information not only has better theoretical properties than the traditional *φ*, as discussed by Mediano *et al*.,^18^ but also has been observed to be superior in practical neuroimaging analyses.^30^

### B Interpretation of Φ

Conceptually, there are several ways to interpret Φ, which highlight different aspects of the systems under study. One inter-pretation—particularly relevant for complexity science -is based on the theory of *information dynamics*, which decomposes information processing in complex systems in terms of storage, transfer, and modification.^31–34^ From this perspective and based on earlier results,^18^ Φ^R^ can be seen as capturing a combination of information modification across multiple parts of the system, information transfer from one part to another, and storage in coherent structures that span across more than one system variable.

Alternatively, a more quantitative and mathematically rigorous way of interpreting Φ^R^ is in terms of *prediction bounds*. The conditional entropy (a complement of the mutual information^35^) provides an upper bound on the optimal prediction performance^36,37^ such that a system with low conditional entropy can be predicted accurately. Therefore, mutual information acts as a bound too: the higher the mutual information between two variables, the better one can be predicted from the other. Thus, Φ^R^ measures to what extent the full state of the system enables better predictions than the states of the parts separately.

Note that other interpretations of Φ-related quantities exist (most notably through information geometry and statistical inference^38,39^), although they do not apply as cleanly to Eqs. (1) and 3).

## III Integrated Information, Metastability, and Phase Transitions

This section explores the usage of integrated information to study dynamical systems, exploring the relationship between Φ, metastability, and phase transitions. For this, we focus on systems of coupled oscillators, which are ubiquitous in both natural and engineered environments, making them of considerable scientific interest.^2^ Typical studies of oscillatory systems—such as the classic work of Kuramoto^40^—examine the conditions under which the system stabilizes on states of either full synchronization or desynchronization, although these two extremes are by no means representative of all real-world synchronization phenomena. Many systems of interest, including the human brain, exhibit synchronous rhythmic activity on multiple spatial and temporal scales but never settle into a stable state, entering so-called chimera-like states^41^ of high partial synchronization only temporarily. A system of coupled oscillators that continually moves between highly heterogeneous states of synchronization is said to be *metastable*.^42^

In 2010, Shanahan^42^ showed that a modular network of phase-lagged Kuramoto oscillators can exhibit metastable chimera states. Variants of this model have since been used to replicate the statistics of the brain under a variety of conditions, including wakeful rest^43^ and anesthesia.^44^ In the following, we study these metastable oscillatory systems through the lens of integrated information theory.

### A Model and measures

**Fig. 2:**
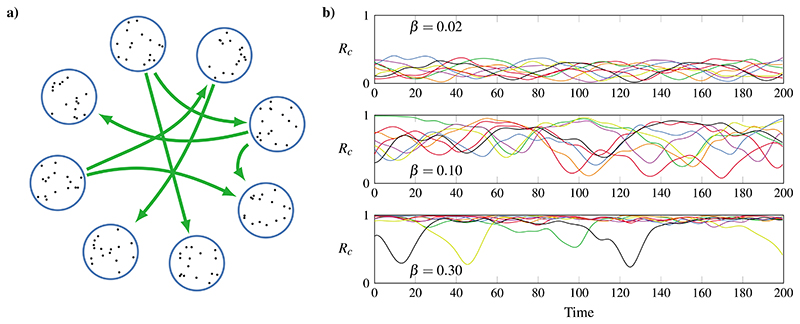
A community-structured network of coupled oscillators exhibits metastable chimera-like behavior. (a) We study a network formed by 8 communities of 32 Kuramoto oscillators each, with strong intra-community coupling and weak inter-community coupling and dynamics parameterized by a phase lag *β*. (b) Time series of synchrony values *R_c_* for each community (*c* = 1, …, 8) show that the behavior of the system changes drastically with *β*. For low *β*, all communities remain desynchronized; for high *β*, all communities synchronize; and it is for intermediate *β* that the system behaves as a metastable chimera, with different communities entering intermittent periods of internal synchrony.

We examine a community-structured network of coupled Kuramoto oscillators (shown in Fig. 2), building on the work of Shanahan.^42^ The network is composed of *N* communities of *m* oscillators each, with every oscillator being coupled to all other oscillators in its community and to each oscillator in the rest of the network with probability *q*. The state of the *i*th oscillator is determined by its phase *θ_i_*, the evolution of which is governed by

(4) $$\frac{d\theta_{i}}{dt}=\omega +\frac{1}{\kappa }\sum_{j}K_{ij}\;\sin\;(\theta_{j}-\theta_{i}-\alpha ),$$

where *ω* is the natural frequency of the oscillators, *α* is a global *phase lag, K_ij_* are the connectivity coefficients, and *κ* is a normalization constant. To reflect the community structure, the coupling between two oscillators *i*, *j* is defined as

(5) $$K_{i,j}=\{\begin{array}{ll} a & \text{if}\;i\;\text{and}\;j\;\text{are}\;\text{in}\;\text{the}\;\text{same}\;\text{community}\;\text{or}\\ b & \text{otherwise,} \end{array}$$

with *a* > *b*. The system is tuned by modifying the value of the phase lag, parameterized by *β* = *π*/2 – *α*. We note that the system is fully deterministic; i.e., there is no noise injected in the dynamical equations.

To assess the dynamical properties of the oscillators, we consider their *instantaneous synchronization R* and *metastability index λ*. The instantaneous synchronization at time *t* of community *c* ∈ {1,…, *N*}, comprising oscillators $I_{c},$ quantifies their dispersion in the *θ*-space,

(6) $$R_{c}(t)=|{\langle e^{i\theta_{j}(t)}\rangle }_{j\in I_{c}}|.$$

Building on this notion, the metastability of each community is defined as the variance of synchrony over time, and the overall metastability is its average across communities,

(7a) $${\text{λ}}_{c}={\mathrm{var}}_{t}R_{c}(t),$$

(7b) $$\lambda ={\langle \lambda_{c}\rangle }_{c}.$$

Communities that are either hypersynchronized or completely desynchronized are both characterized by small values of λ_*c*_, whereas only communities whose elements fluctuate in and out of synchrony have a high λ_*c*_. Put simply, a system of oscillators exhibits metastability to the extent that its elements fluctuate between states of high and low synchrony. In addition to metastability, we also consider the *global synchrony* of a network defined as the spatiotemporal average of the instantaneous synchrony,

(8) $$\xi =\langle R_{c}(t)\rangle_{t,c}.$$

For tractability, we calculate Φ^R^ with respect to the *coalition configuration* of the system, defined for each community *c* and time *t* as

$$X_{t}^{c}=\{\begin{array}{ll} 1 & \text{if}\;R_{c}(t)\;>\;\gamma ,\\ 0 & \text{otherwise,} \end{array}$$

where *γ* is the coalition threshold. This representation provides *N* interdependent binary time series $X_{t}≔(X_{t}^{1},.\;.\;.\;,\;X_{t}^{N}),$ which indicates the set of communities that are more internally synchronized. The Shannon entropy of *X_t_* is referred to as the *coalition entropy H_c_* and quantifies the diversity of synchronization patterns across communities.

### B Results

We simulated a network composed of *N* = 8 communities of *m* = 32 oscillators each. The probability of connections across communities was set to *q* = 1/8, with connection strengths of *a* = 0.6 within communities and *b* = 0.4 across. The natural frequency used was *ω* = 1 and the normalization constant *κ* = 64. We ran 1500 simulations with values of *β* distributed uniformly at random in the range [0, 2*π*) using a 4th-order Runge-Kutta algorithm, using a step size of 0.05 for numerical integration. Each simulation was run for 5 × 10^6^ time steps, discarding the first 10^4^ to avoid transient effects and applying a thinning factor of 5. For the results presented here, we used *γ* = 0.8, and we confirmed that results were qualitatively stable for a wide range of threshold values. All information-theoretic measures are reported in bits.

#### 1 Metastability and Φ^R^ at the phase transition

**Fig. 3:**
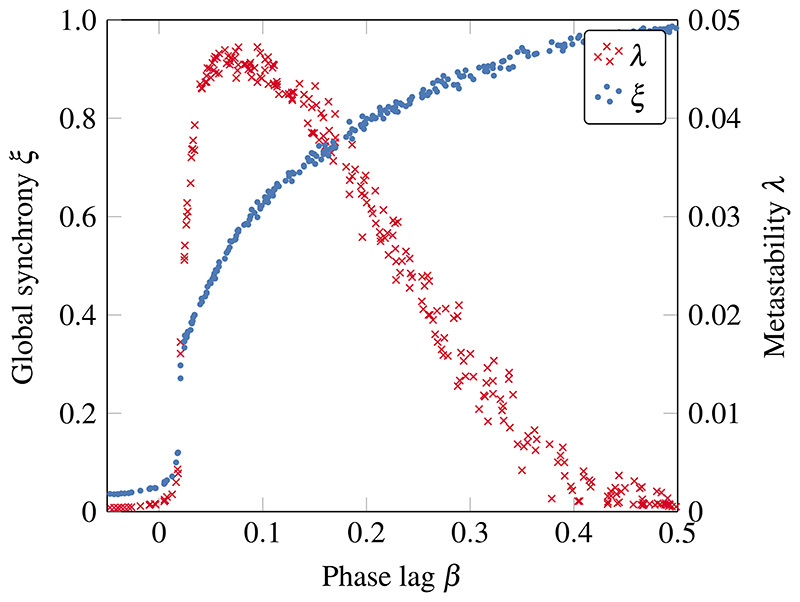
Phase transition between desynchronization and hypersynchronization in coupled oscillators. Global synchrony *ξ* and metastability *λ* for different phase lags *β* in the critical region. Rapid increase of metastability marks the onset of the phase transition.

We first study the system from a purely dynamical perspective, and, replicating previous findings,^42^ we find two well differentiated dynamical regimes: one of hypersynchronization and one of complete desynchronization, with strong metastability appearing in the narrow transition bands between them (Fig. 3). Interestingly, it is in this transition region where the oscillators operate in a critical regime poised between order and disorder and where complex phenomena appear. As the system moves from desynchronization to full synchronization, there is a sharp increase in metastability, followed by a smoother decrease as the system becomes hypersynchronized.

**Fig. 4:**
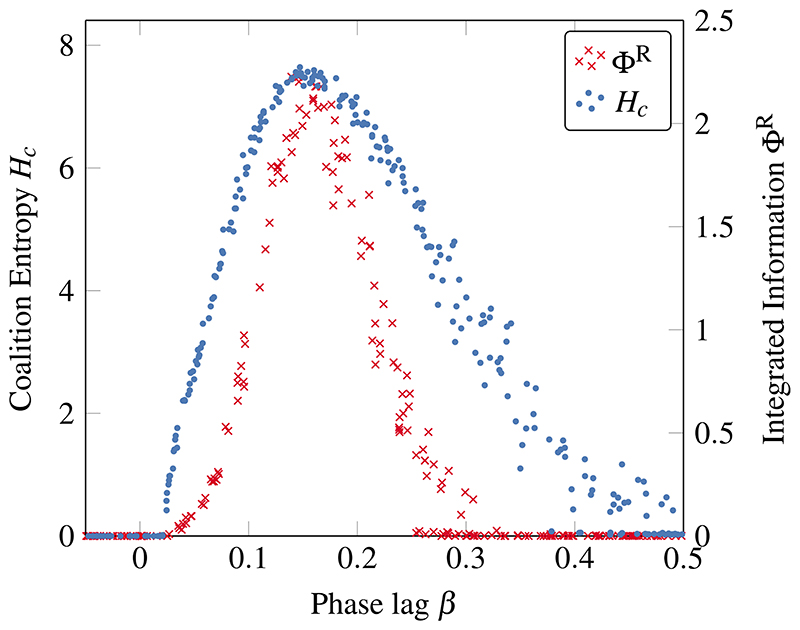
Integrated information Φ^R^ peaks in the phase transition of coupled oscillators. Within the broad region between order and disorder in which *H_c_* rises, there is a narrower band in which complex spatiotemporal patterns generate high Φ^R^. Calculations use *τ* = 100.

Importantly, Φ^R^ was found to exhibit a similar behavior to λ: it is zero for desynchronized systems, peaks in the transition region, and shrinks again in the fully ordered regimes (Fig. 4). This shows that networks in metastable regimes are the only ones that exhibit integrated information. When comparing Φ^R^ with the coalition entropy, results show both peaks at the same point, although the peak in Φ^R^ is much narrower than the peaks in λ and *H_c_*. Hence, while some values of *β* do give rise to non-trivial dynamics, it is only at the center of the critical region that these dynamics give rise to integrated information.

These results imply that Φ^R^ is sensitive to more subtle dynamic patterns than the other measures considered and is in that sense more discriminating. In effect, a certain degree of internal variability is necessary to establish integrated information, but not all configurations with high internal variability lead to a high Φ^R^. Also, Φ^R^ accounts for spatial *and* temporal patterns in a way that the other metrics do not.^45^

#### 2 Integrated information at multiple timescales

**Fig. 5:**
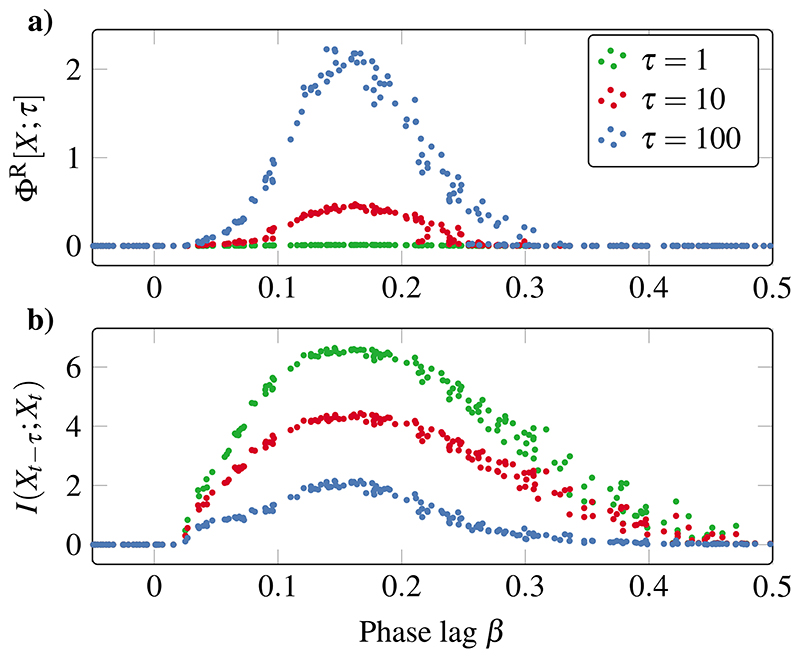
Redundancy dominates at shorter timescales, synergy at longer ones. (a) Integrated information Φ^R^ grows with increasing integration timescale *τ*, indicating a shift from redundancy-to synergy-dominated dynamics. (b) Time-delayed mutual information *I*(*X_t–τ_; X_t_*) decreases with higher *τ*, indicating an overall loss of predictability for longer time horizons.

As a further analysis, we can investigate the behavior of Φ^R^ at multiple timescales by varying the integration timescale parameter *τ* (see Fig. 2 for a visual guide of different *τ* values). Figure 5 shows Φ^R^ for several values of *τ* and compares it with standard time-delayed mutual information (TDMI) *I*(*X_t-τ_*; *X_t_*). Note that this analysis cannot be carried out with *H_c_* or other measures of complexity that are not sensitive to temporal order—i.e., that are functions of *p*(*X_t_*) and not *p*(*X_t_*\*X_t-τ_*).

Results show that Φ^R^ and TDMI exhibit opposite trends with respect to changes in *τ*: TDMI decreases with higher *τ*, while Φ^R^ increases. At short timescales, the system is highly predictable—thus the high TDMI—but this short-term evolution does not imply much system-wide interaction—thus the low Φ^R^. Together, the high TDMI and low Φ^R^ suggest that at short timescales, the system is redundancy-dominated: the system contains information about its future, but this information can be obtained from the parts separately. Conversely, for prediction at longer timescales, TDMI decreases but Φ^R^ increases, indicating that while the system is overall less predictable, this prediction is enabled by the information contained in the interaction between the parts.

#### 3 Robustness of Φ^R^ against measurement noise

**Fig. 6:**
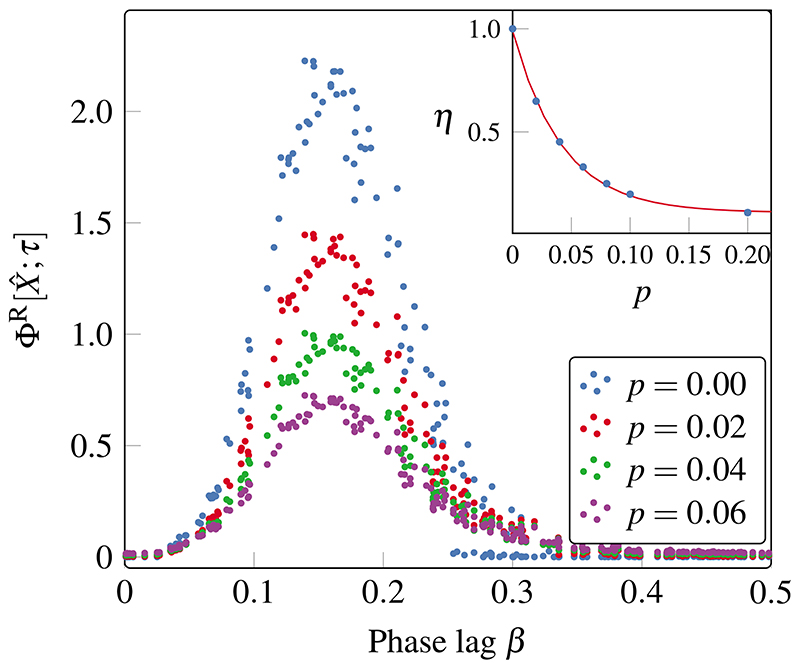
Integrated information is highly sensitive to measurement noise. Integrated information Φ^R^ for different levels of measurement noise *p*. The inset shows the mean and variance of the ratio *η* between Φ^R^ of the corrupted and the original time series (blue) and an exponential fit *η* = exp(–*p*/*ℓ*), with *ℓ* ≈ 0.04 (red).

Finally, we study the impact of measurement noise on Φ^R^, wherein the system runs unchanged, but our recording of it is imperfect. For this, we run the (deterministic) simulation as before and generate the sequence of coalition configurations and then emulate the effect of uncorrelated measurement noise by flipping each bit in the time series with probability *p*, yielding a corrupted time series ${\hat{X}}_{t}.$ Finally, Φ^R^ is recalculated on the corrupted time series (Fig. 6). To quantify the impact of noise, we studied the ratio between the corrupted and the original time series,

(9) $$\eta =\frac{\Phi^{\text{R}}[\hat{X};\;\tau ]}{\Phi^{\text{R}}[X;\;\tau ]}.$$

To avoid instabilities as Φ^R^[*X; τ*] ≈ 0, we calculate *η* only in the region within 0.05 rad of the center of the peak shown in Fig. 4, where Φ^R^[*X; τ*] is large. The inset of Fig. 6 shows the mean and standard deviation of *η* at different noise levels.

Results show that Φ^R^ decays exponentially with *p* (Fig. 6, upper panel), reflecting a gradual loss of the precise spatiotemporal patterns that are characteristic of the system. In particular, Φ^R^ was found to be highly sensitive to noise and to undergo a rapid decline, as a measurement noise of 5% can wipe out 70% of the observed integrated information of the system. While the distortion has a stronger effect on time series with greater Φ^R^, it preserves the dominant peak for all values of *p*.

Overall, in this section, we have shown that a network of Kuramoto oscillators presents a sharp, clear peak of integrated information around its phase transition that coincides with a strong increase in metastability. Furthermore, we have found that Φ^R^ is informative, as it can reveal information about timescales of interaction between system components. Finally, Φ^R^ was also found to be sensitive, as it vanishes quickly if the specific spatiotemporal patterns of the system under study are disrupted. This, in turn, suggests that it is highly unlikely to observe significant values of Φ^R^ due to artifacts induced by (uncorrelated observational) noise.

## IV Integrated Information and Distributed Computation

In Sec. III, we related integrated information to dynamical complexity by linking Φ^R^ with criticality and metastability in coupled oscillators. We now move on to cellular automata (CA), a well-known class of systems widely used in the study of distributed computation.^31,46^ Our aim here is to relate IIT to distributed computation in two ways: at a global scale, Φ^R^ is higher for complex, class IV^13^ automata and at a local scale Φ^R^ is higher for emergent coherent structures, such as blinkers, gliders, and collisions.

A CA is a multi-agent system in which every agent has a finite set of possible states and evolves in discrete time steps following a set of simple rules based on its own and other agents’ states. CA have been often used in studies of self-organization,^1,47^ and some of them are capable of universal computation.^13^ In a CA, agents (or *cells*) are arranged in a one-dimensional cyclic array (or *tape*). The state of each cell at a given time step has a finite number of possible states, which is determined via a boolean function (or *rule*), which uses as arguments the state of itself and its immediate neighbors at the previous time step. The same boolean function dictates the evolution of all agents in the system, inducing a spatial translational symmetry. Each CA, irrespective of its number of agents, is usually denoted by its rule.^1^

For all the results presented below, we follow the simulation parameters used by Lizier in his study of local information dynamics in CA:^31^ we initialize a tape of length 10^4^ with i.i.d. random variables, discard the first 100 steps of simulation, and run 600 more steps that are used to estimate the probability distributions used in all information-theoretic measures.

### A Integrated information and complexity classes

Our first analysis focuses on elementary cellular automata (ECA), a specific subclass of CA. In ECA, each cell has two possible states (usually denoted as white or black). ECA are traditionally denoted by their rule number, between 0 and 255, and grouped in four complexity classes:^13^ Class I rules have attractors consisting of single absorbing states; Class II rules evolve toward periodic orbits with relatively short cycle lengths; and Class III and IV rules have attractors with length of the order of the size of their phase space, with the latter being characterized by the presence of highly structured patterns and persistent structures.

**Fig. 7:**
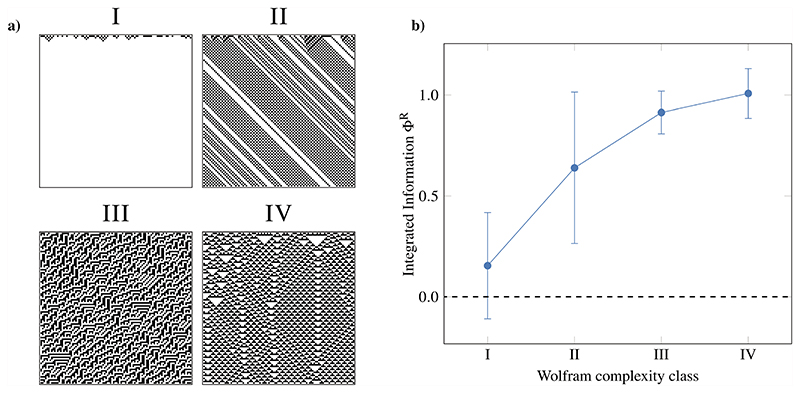
Integrated information grows monotonically with Wolfram class number. (a) Examples of each complexity class (ECA rules 32,56, 75, and 54, respectively), showing noticeable differences in behavior. Notice the presence of localized particles in the class IV rule. (b) Correspondingly, Φ^R^ is the highest for the more complex classes IV and III and lower (and often negative) for the simpler behaviors in classes I and II (error bars correspond to standard deviation across rules; each rule was simulated multiple times to obtain an accurate estimation of Φ^R^).

As a first experiment, we calculate the average integrated information of each ECA, separating each automaton by complexity class (Fig. 7). For this, we followed the classifications defined in Wolfram’s original article^13^ as well as other clear-cut rules and excluded border cases, which did not neatly fit into a single category.

Results show that Φ^R^ correlates strongly with complexity as discussed by Wolfram: automata of higher classes have consistently higher Φ^R^ than automata of lower classes, and the difference between classes I, II and III, IV is stark.

It is worth noting the small difference between classes III and IV. This is likely related to the blurriness of the line separating both classes—visually, it is hard to judge whether structures are “coherent enough” to support distributed computation, and formally, the problem of determining whether a particular rule belongs to class III or IV is considered undecidable.^48,49^ Based on this, we may tentatively suggest that the capacity to integrate information is a necessary, but not sufficient, condition for universal computation.

### B Integrated information at the edge of chaos

In his seminal 1990 article, Langton^14^ took a step beyond Wolfram’s classification and argued that the complexity and universality observed in ECA may reflect a broader phenomenon called *computation at the edge of chaos*. In this view, computation is made possible by indefinitely long transient states, a manifestation of *critical slowing-down*,^50^ that form the particle-like structures seen in class IV rules.

**Fig. 8:**
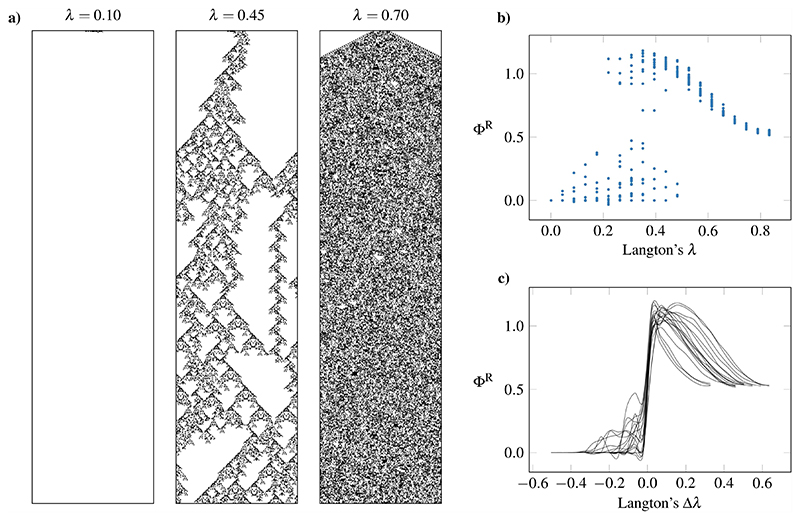
Integrated information peaks at the edge of chaos. (a) Sample runs from a random 6-color, range-2 cellular automaton with different *λ* values, starting from a blank tape with 20 randomized cells in the middle. The parameter *λ* corresponds to the fraction of non-blank cells in an automaton’s update rule. (b) Integrated information Φ^R^ peaks at an intermediate level of *λ*. (c) When plotted against Δ*λ*, the distance from a transition event, all runs align on a similar Φ^R^ profile.

Langton’s argument starts by defining a parameter λ, which represents the fraction of neighborhoods in a CA’s rule table that map to a non-quiescent state (i.e., a non-white color). Then, by initializing one automaton with an empty rule table and progressively filling it with non-quiescent states, one can observe a transition point with exponentially long, particle-like transients [Fig. 8(a)]. Here, we repeat Langton’s experiments using a 6-color, range-2 CA, and compute its average Φ as its rule table gets populated and λ increases.

In agreement with Langton’s argument, we found that integrated information has largest values for intermediate values of λ, coinciding with the automata’s transition to a chaotic regime [Fig. 8(b)]. Interestingly, this shows that rules with high Φ^R^ are the ones at the critical region—where computation is possible.

Another unusual feature of Fig. 8(b) is that there is a region where complex, high-Φ^R^ automata coexist with simpler ones. This phenomenon was reported by Langton^14^ already: different automata experience a “transition to chaos” at different values of λ. This motivated a further analysis of measures of complexity as a function of Δλ, the distance from the transition event for that particular automaton. As expected, when aligned by their critical λ value and plotted against Δλ [Fig. 8(c)], all curves align onto a consistent, seemingly universal, picture of integrated information across the λ range.

For completeness, it is worth mentioning why at the right side of Figs. 8(b) and 8(c), Φ^R^ does not vanish for high λ (as one could expect, given that the single-cell autocorrelation does^14^). This is essentially due to the determinism and locality of the automaton’s rule: given a spatially extended set of cells, it is always possible to predict the middle ones with perfect certainty. At the same time, cutting the system with a bipartition will reduce the spatial extent of this predictable region so that the predictability of the whole is greater than the predictability of the parts, and thus, Φ > 0.

### C Information is integrated by coherent structures

In the experiments above, we have shown that more complex automata integrate more information. However, this is not enough to make a case for Φ^R^ as a marker of distributed computation—it may just be the case that medium-λ CA have higher Φ^R^ due to general properties of their rule tables or for some other reasons. In this section, we address this possible counter-argument by showing that the increase in Φ^R^ is due to the emerging particles and, therefore, can be directly associated with distributed computation.

**Fig. 9:**
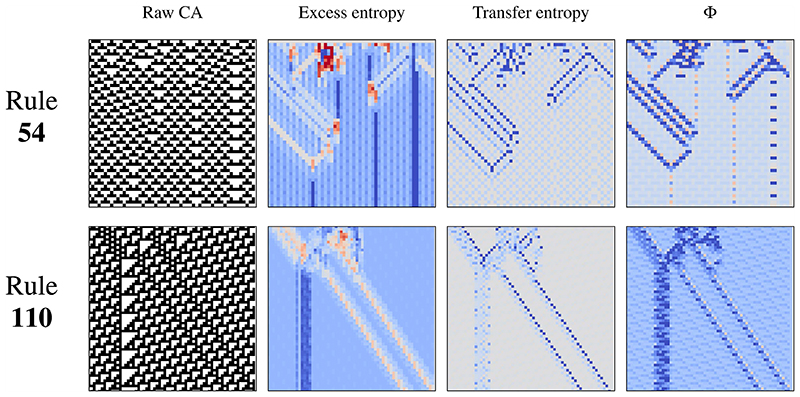
Local integrated information detects coherent structures in cellular automata. Local information measures in cellular automata, applied to simulations of rule 54 (top) and 110 (bottom). Local excess entropy is high for static particles, local transfer entropy for moving particles, and local integrated information Φ for both. For all these measures, blue and red indicate positive and negative values, respectively.

To show this, we run large simulations of ECA rules 54 and 110 and evaluate several local information measures in small fragments of both (Fig. 9). Specifically, we compute Lizier’s measures of storage (excess entropy, *e_k_*) and transfer (transfer entropy, *t_k_*), as well as local integrated information, using the binary time series of local neighborhoods of ECA cells as input (details in the Appendix). Note that this pointwise Φ has not been ΦID-revised, as the development of pointwise ΦID metrics is still an open problem.^51^ A dedicated treatment of pointwise ΦID is part of our ongoing work and will be presented in a separate publication.

As expected, TE is high in gliders (moving particles), while excess entropy is high in blinkers (static particles), confirming Lizier’s results that these structures perform information transfer and storage in CA.^31^ More interestingly for our purposes is that Φ is high in *all of them*—gliders, blinkers, and the collisions between them.

When studied at a local scale in space and time, we see that information integration encompasses the three categories—storage, transfer, and modification—and that Φ can detect all of them *without having been explicitly designed to do so*. This reinforces our claim that Φ (and, in turn, its revised version Φ^R^) is a generic marker of emergent dynamics and is connected with known measures of information processing. This relationship can be understood in a more mathematically rigorous manner via ΦID,^18^ which shows that Φ is decomposable in terms of information transfer and synergy.

## V Discussion

Many theoretical efforts to understand complexity have their roots in information-theoretic or dynamical systems perspectives. On the one hand, information-theoretic approaches focus on discrete systems displaying coordinated activity capable of universal computation, e.g., via particles in Wolfram’s class IV automata.^13^ On the other hand, dynamical approaches focus on continuous coupled dynamical systems near a phase transition, displaying a stream of transient states of partial coherence that balance robustness and adaptability.^52,53^ The results presented in this paper reveal how integrated information is effective at characterizing complexity in both information-processing and dynamical systems and hence can be regarded as a common signature of complex behavior across a wide range of systems. Overall, this paper shows how a grounded, demystified understanding of IIT combined with the improved metrics provided by ΦID can be used as a first step toward a much needed point of contact between these diverging branches of complexity science, helping to bridge the gap between information-processing and dynamical approaches.

### A On the relationship between integrated information and metastability, criticality, and distributed computation

It is important to remark that the relationship between integrated information, metastability, criticality, and distributed computation is not an identity and that their agreement is an important finding conveyed by Φ.

Metastability, in the case of oscillator networks, is a community-local quantity—that is, it corresponds to an average over quantities (λ_*c*_) that depend on the temporal diversity seen within each community, independently of the rest. In stark contrast, Φ relies on the irreducible interactions between communities. Interestingly, our finding reveals a close relationship between the two, insofar as internal variability enables the system to visit a larger repertoire of states in which system-wide interaction can take place.

Criticality is, in general, enabled by a precise balance of two opposite forces (typically characterized as order and chaos in physics) that enables peculiar and fascinating phenomena, such as scale-freeness, extreme sensitivity to perturbations, and universality classes.^54,55^ In contrast, one of the core ideas in IIT is that integration and differentiation are not opposite forces but can actually coexist together.^56,57^ Therefore, integrated information is not maximized by optimal trade-offs, but by mechanisms that incentivize direct and synergistic information transfer.^18^

Finally, distributed computation is mainly based on intuitive but ultimately informal notions developed after Wolfram’s and Langton’s work. Our results establish a strong relationship between the capability of information-processing complex systems to integrate information and their ability to perform highly non-trivial information processing via emergent coherent structures, such as blinkers, gliders, and collisions. The fact that higher integrated information was carried by coherent emerging structures is consistent with recent accounts of *causally emergent dynamics*,^58^ which further supports the case of Φ being an effective quantitative indicator of distributed emergent computation with numerous potential future applications.

### B Related work

The conceptual link between complexity and integrated information is by no means a novel idea: in fact, in the early days of IIT, integrated information and complexity were closely intertwined^59^—as was its close relation with the theory of complex networks.^60^ Unfortunately, as the theory turned more convoluted and less applicable, this link lost relevance and IIT drifted away from standard information-theoretic methods.

Nonetheless, recent research has again brought complexity science to the fore within the IIT community. In some cases, information-theoretic measures originally conceived as measures of complexity have been re-purposed within IIT.^61^ In others, new measures of complexity are inspired by previous work on IIT.^39^ In contrast with its consciousness-focused sibling, advancements in this more pragmatic IIT (cf. Sec II) have been enabled by a simplification and unification of the underlying principles of the theory—for example, in terms of information geometry^62^ or information decomposition.^18^

Given its origins in theoretical neuroscience, it is no surprise that most of IIT’s relevance to complex systems has been mostly on models of neural dynamics. In this context, the combination of integration and segregation captured by Φ has been linked to other dynamical features of interest (such as metastability and neural avalanches) in a variety of settings, including, e.g., models of whole-brain activity^63^ and spiking neural networks.^64^

Relatedly, there has been recent work linking Φ with phase transitions in thermodynamic systems.^65^ Together with recent results linking information processing and stochastic thermodynamics,^66^ this exciting avenue of research opens the door for a purely physical interpretation of Φ in general physical systems.

Finally, note that for our main analyses, we estimate Φ^R^ using the simple yet effective MMI redundancy function.^29^ However, there are multiple possible redundancy functions,^67,68,74^ and while they tend to yield qualitatively similar results in practice,^30,69^ they have been shown to differ in some specific scenarios,^68^ and therefore, it is important to elucidate their similarities and differences in practical analyses. There is ongoing investigation into the different properties of each redundancy function, and future work will explore the specific advantages of diverse redundancy functions when used to calculate Φ^R^.

### C Concluding remarks

This paper puts forward a pragmatic argument that metrics of integrated information—such as the ones provided by IIT and ΦID—can allow us to investigate unexplored commonalities between informational and dynamical complexity, pointing out a promising avenue to reconcile their divide and benefit subsidiary disciplines, such as cognitive and computational neuroscience. Note that Φ is by no means the only quantity that peaks with the system’s complexity—in cellular automata, one could use the autocorrelation of a single cell and in coupled oscillators the variance of Kuramoto’s order parameter. However, the feature that makes Φ unique is that *it is applicable across the board* and yields the desired results in different kinds of systems without requiring idiosyncratic, *ad hoc* measures. This unification, analogous to the transversal role that Fisher information plays in phase transitions over arbitrary order parameters,^70^ posits integrated information as a key quantity for the study of complex dynamics, whose relevance has just started to be uncovered.

## Supplementary Material

Appendix

## Data Availability

Data sharing is not applicable to this article as no new data were created or analyzed in this study.

## Box

Originally conceived within theoretical neuroscience, integrated information theory (IIT) has been rarely used in other fields—such as complex systems or non-linear dynamics—despite the great value it has to offer. In this article, we inspect the basics of IIT, dissociating it from its contentious claims about the nature of consciousness. Relieved of this philosophical burden, IIT presents itself as an appealing formal framework to study complexity in biological or artificial systems, applicable in a wide range of domains. To illustrate this, we present an exploration of integrated information in complex systems and relate it to other notions of complexity commonly used in systems such as coupled oscillators and cellular automata. Through these applications, we advocate for IIT as a valuable framework capable of revealing common threads between diverging branches of complexity science.
