# Supplementary material for: Integrated information as a common signature of dynamical and information-processing complexity
Source: Chaos. Author manuscript; Available in PMC 2023 Jul 18. (PMC7614772; doi:10.1063/5.0063384)
Supplement: Appendix [file EMS177459-supplement-Appendix.pdf]

APPENDIX: LOCAL INTEGRATED INFORMATION

A natural way to extend IIT 2.0 for complex systems analysis is to consider local versions of Φ, which can be built via the framework introduced by Lizier.[32] Local (or pointwise) information measures are able to identify coherent, emergent structures known as *particles*, which have been shown to be the basis of the distributed information processing that takes place in systems such as cellular automata.[31,33,34]

One of the most basic pointwise information metrics is the local mutual information, which is defined as

$$i(x;y) := \log \frac{p(x,y)}{p(x)p(y)} \tag{A1}$$

so that $\mathbb{E}[i(X;Y)] = I(X;Y)$ is the usual mutual information. By evaluating i on every x, y pair, one can determine which particular combinations of symbols play a predominant role for the observed interdependency between X and Y. (More specifically, the local mutual information captures specific deviations between the joint distribution and the product of the marginals.) Building on these ideas, Lizier proposed a taxonomy of distributed information processing as composed of *storage*, *transfer*, and *modification*.[31] For this, consider a bivariate stochastic process (X_t, Y_t) with $t \in \mathbb{Z}$ and introduce the shorthand notation $X_t^{(k)} = (X_{t-k}, \ldots, X_t)$ and $X_t^{(k^+)} = (X_t, \ldots, X_{t+k-1})$ for the corresponding past and future embedding vectors of length k. In this context, storage within the subprocess X_t is identified with its *excess entropy* $\mathrm{E}_k = I(X_t^{(k)}; X_{t+1}^{(k^+)})$[71] and transfer from Y_t to X_{t+1} with the *transfer entropy* $\mathrm{TE}_k = I(X_{t+1}; X_t^{(k)} | Y_t^{(k)})$.[72] Interestingly, both quantities have corresponding local versions,

$$e_k(x_t) := \log \frac{p(x_t^{(k)}, x_{t+1}^{(k^+)})}{p(x_t^{(k)})p(x_{t+1}^{(k^+)})}, \tag{A2}$$

$$t_k(y_t \to x_t) := \log \frac{p(x_{t+1}|x_t^{(k)}, y_t^{(k)})}{p(x_{t+1}|x_t^{(k)})} \tag{A3}$$

such that, as expected, $\mathbb{E}[e_k] = \mathrm{E}_k$ and $\mathbb{E}[t_k] = \mathrm{TE}_k$. Note that to measure transfer in either direction for the results in Fig. 9, we compute the local TE from a cell to its left and right neighbors and take the maximum of the two.

These ideas can be used to extend the standard formulation of integrated information measures in two ways. First, by using embedding vectors, the IIT metrics are applicable to non-Markovian

systems.[73] Second, by formulating pointwise measures, one can capture spatiotemporal variations in Φ. Mathematically, we reformulate Eq. (2) introducing these modifications as

$$\varphi_k[X;\tau,\mathcal{B}] = I(X^{(k)}_{t-\tau};X_t) - \sum_{j=1}^{2} I(M^{j,(k)}_{t-\tau};M^j_t) \tag{A4}$$

and apply the same partition scheme described in Sec. II A to obtain an "embedded" integrated information, Φ_k. Then, the equation above can be readily made into a local measure by replacing mutual information with its local counterpart,

$$\phi_k[x_t;\tau,\mathcal{B}] = i(x^{(k)}_{t-\tau};x_t) - \sum_{j=1}^{2} i(m^{j,(k)}_{t-\tau};m^j_t) \tag{A5}$$

such that, as expected, $\varphi_k[X;\tau,\mathcal{B}] = \mathbb{E}\left[\phi_k[x_t;\tau,\mathcal{B}]\right]$.
